# Murine Es-derived cardiomyocytes form grafts and improve cardiac function in the infarcted myocardium

**DOI:** 10.1186/1532-429X-11-S1-P174

**Published:** 2009-01-28

**Authors:** Hualei Zhang, Hui Qiao, Satoshi Yamanaka, Nataliya Petrenko, Vickas Patel, Bin Huang, Victor Ferrari, Kenneth Boheler, Rong Zhou

**Affiliations:** 1 University of Pennsylvania, Philadelphia, PA USA; 2 National Institute of Aging, NIH, Bethesda, MD USA

**Keywords:** Embryonic Stem Cell, Embryonic Stem Cell Line, Murine Embryonic Stem Cell, Vehicle Treated Animal, Global Cardiac Function

## Background

embryonic stem cells (ESC) readily differentiate into cardiac lineage making them a potential source of transportable cells for myocardial regeneration. However the low yield of ESC-derived cardiomyocytes (ESC-CMs) using the conventional differentiation method makes it difficult to perform *in vivo* study and low enrichment of CMs leads to concerns of teratoma formation.

## Methods

a murine ESC line containing puromycin resistance gene under control of a cardiac specific promoter, sodium calcium exchanger (NCX) was used to generate ESC-CMs. ESC-CMs were labeled with Superparamagnetic iron-oxide nanoparticles (SPIO) for MRI detection. Reperfused myocardial infarction was induced in athymic rats. Infarction size was estimated by MRI post-op day1 to exclude animals with infarct size smaller than 10% or larger than 35%of the LV volume. At post-op day 7, labeled ESC-CMs (5–10 millions) were injected into infarction region. Control group was injected with vehicle. MRI scan was performed at post-op day 8 to confirm successful CM cell transplantation. Global cardiac function in ESC-CM and vehicle treated animals was assessed by MRI for 2 months. Immunohistology staining and electrophysiology were performed on postmortem hearts and ESC-CMs, respectively.

## Results

**Figure 1:**
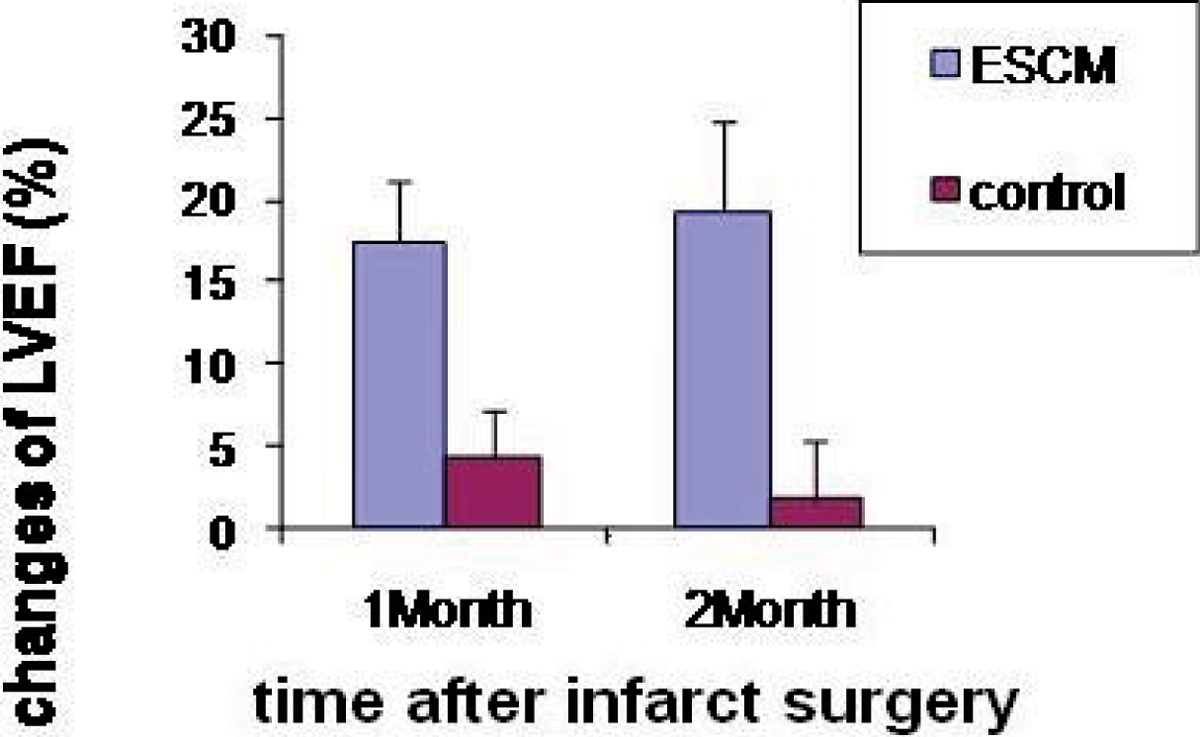
**Left-ventricular ejection fraction LVEF changes**. The rats treated with ESCM showed much higher the increase of LVEF change than the control rats both at 1 month (p = 0.0003) and 2 months (p = 0.000048), while the infarction size at the day 1 post-op was no significant difference between two groups (20.02 ± 3.11 v.s 19.09 ± 4.72, p = 0.65).

a high yield of ESC-CMs was achieved with positive cardiac specific alpha-actinin in more than 90% of cells. The low proliferative capacity of ESC-CMs allows them to retain SPIO for serial MRI tracking. LV ejection fraction in ESC-CM treated rats at 1- and 2-month is significantly higher than that in the controls. IHC demonstrated formation of grafts in the host myocardium and gap junctions between grafted ESC-CMs and host CMs. See Figure 1.

## Conclusion

Large numbers of highly pure ESC-CMs were obtained. Preliminary results suggest that ESC-CMs form grafts and improve LV function in the infarcted hearts.

